# Tight correlation between expression of the Forkhead transcription factor FOXM1 and HER2 in human breast cancer

**DOI:** 10.1186/1471-2407-8-42

**Published:** 2008-02-06

**Authors:** Nuran Bektas, Anette ten Haaf, Jürgen Veeck, Peter Johannes Wild, Juliane Lüscher-Firzlaff, Arndt Hartmann, Ruth Knüchel, Edgar Dahl

**Affiliations:** 1 Institute of Pathology, University Hospital of the RWTH Aachen, Aachen, Germany; 2 Institute of Pathology, University Hospital Zürich, Zürich, Switzerland; 3 Institute of Biochemistry, University Hospital of the RWTH Aachen, Aachen, Germany; 4 Department of Pathology, University of Erlangen, Erlangen, Germany

## Abstract

**Background:**

FOXM1 regulates expression of cell cycle related genes that are essential for progression into DNA replication and mitosis. Consistent with its role in proliferation, elevated expression of FOXM1 has been reported in a variety of human tumour entities. *FOXM1 *is a gene of interest because recently chemical inhibitors of FOXM1 were described to limit proliferation and induce apoptosis in cancer cells *in vitro*, indicating that FOXM1 inhibitors could represent useful anticancer therapeutics.

**Methods:**

Using immunohistochemistry (IHC) we systematically analysed FOXM1 expression in human invasive breast carcinomas (n = 204) and normal breast tissues (n = 46) on a tissue microarray. Additionally, using semiquantitative realtime PCR, a collection of paraffin embedded normal (n = 12) and cancerous (n = 25) breast tissue specimens as well as benign (n = 3) and malignant mammary cell lines (n = 8) were investigated for FOXM1 expression. SPSS version 14.0 was used for statistical analysis.

**Results:**

FOXM1 was found to be overexpressed in breast cancer in comparison to normal breast tissue both on the RNA and protein level (e.g. 8.7 fold as measured by realtime PCR). We found a significant correlation between FOXM1 expression and the HER2 status determined by HER2 immunohistochemistry (*P *< 0.05). Univariate survival analysis showed a tendency between FOXM1 protein expression and unfavourable prognosis (*P *= 0.110).

**Conclusion:**

FOXM1 may represent a novel breast tumour marker with prognostic significance that could be included into multi-marker panels for breast cancer. Interestingly, we found a positive correlation between FOXM1 expression and HER2 status, pointing to a potential role of FOXM1 as a new drug target in HER2 resistant breast tumour, as FOXM1 inhibitors for cancer treatment were described recently. Further studies are underway to analyse the potential interaction between FOXM1 and HER2, especially whether FOXM1 directly activates the *HER2 *promoter.

## Background

Fox (Forkhead Box) proteins, characterised by a 100 amino acid winged-helix DNA binding domain, are chordate transcription factors that play important roles in the regulation of growth and development [1,2]. FOXM1 is ubiquitously expressed in cells undergoing proliferation [3,4]. It is required for normal coupling of DNA replication (at S phase) and chromosomal segregation (at M phase) during cell cycle progression [5]. FOXM1 expression peaks at G2/M-transition and is believed to exert its S-M coupling role by promoting M phase entry and suppressing DNA re-replication [6].

FOXM1 is localised mainly in the cytoplasm during late G1 and S phases, but is found to be phosphorylated and translocated to the nucleus before cells entry into the G2/M phase. Activation of the Raf/MEK/MAPK pathway is necessary for the nuclear translocation of FOXM1 protein [6]. Consistent with its role in promoting proliferation, elevated expression of FOXM1 has recently been reported in a variety of human tumour entities including breast [7] and liver cancer [8]. FOXM1 depletion causes a certain form of cell death – so called mitotic catastrophe – that occurs during mitosis often arising from aberrant G2 checkpoint control [9]. Therefore inhibition of FOXM1 expression could represent a new target in the therapeutic treatment of breast cancer [9].

Very recently, *in vitro *data have demonstrated a direct regulation of the oestrogen receptor gene (*ESR1*) by FOXM1 protein binding to the *ESR1 *promoter, thus leading to upregulation of oestrogen receptor-alpha (ERα) expression at mRNA transcript and protein levels [10]. It is well known that oestrogen receptors play a major role in the regulation of growth, survival and differentiation of normal and malignant breast epithelial cells [11,12]. Therefore the determination of breast tumour hormone receptor status is of major importance for therapy selection [13]. Approximately 60–80% of all breast cancers abundantly express ERα, but only two thirds of those patients are responsive to endocrinal treatment (anti-oestrogen therapy). Intriguingly, a proportion of ERα-positive tumours do not respond to hormone treatment at all (*de novo *resistance) whilst the majority of those tumours that initially responded to anti-oestrogens eventually become resistant during treatment (acquired resistance). Most ER-resistant tumours remain ERα-positive, suggesting a continued role for ERα in breast cancer cell survival and proliferation [14,15]. The very likely development of ER-resistance during breast cancer treatment with anti-oestrogens, like the resistances described for treatment with the HER2 antibody Herceptin^®^, emphasises that there is an urgent need for surrogate target molecules that may allow bypassing these resistances. Recently, numerous candidate genes which are highly differentially expressed in breast cancer have been identified by our research group [16,17]. *FOXM1 *is one of these candidate genes and has been selected for further analysis because chemical inhibitors of this target molecule are already available and have been shown to limit proliferation e.g. in liver cancer cells *in vivo *[18,19]. Thus FOXM1 inhibitors could represent useful new anticancer therapeutics for breast cancer as well. So far little is known about the expression of FOXM1 in normal and cancerous human breast tissue. In the present study we systematically analysed the expression of FOXM1 in human breast carcinomas and normal breast tissue on both the mRNA and protein level and analysed the results especially in correlation to hormone receptor status, HER2 status and patient survival data.

## Methods

### Patient samples on the breast cancer tissue microarray (TMA)

FOXM1 protein expression in breast cancer patients was assessed using a TMA that has been previously described [17] and that consisted of 204 breast cancer specimens and 46 normal breast tissue specimens. The TMA contained one tissue core from non-selected, formalin-fixed and paraffin-embedded primary breast cancer specimens diagnosed between 1994 and 2002 at the Institute of Pathology, University of Regensburg, Germany. Patients' age ranged from 25 to 82 years with a median age of 56 years. An experienced surgical pathologist (A.H.) evaluated H&E-stained slides of all specimens prior to construction of the TMA in order to identify representative tumour areas. Histologically, all tumours were graded according to Elston and Ellis [20]. Clinical follow-up data, provided by the Central Tumour Registry, Regensburg, Germany were available for all 204 breast cancer patients with a median follow-up period of 78 months (range 0–148 months). All patients gave informed consent for retention and analysis of their tissue for research purposes and the Institutional Review Board of the participating centre approved the study. The paraffin-embedded and formalin-fixed normal (n = 14) and cancerous (n = 25) breast tissue samples for mRNA expression analysis have been described previously [21].

### Cell lines

The human mammary epithelial cell lines HMEC, MCF10A and MCF12A as well as the breast cancerous cell lines MCF7, T47D, ZR75-1, MDA-MB231, MDA-MB468, MDA-MB435s, SKBR3 and BT20 were obtained from the ATCC (Rockville, MD, USA) and cultured as previously described [22].

### RNA extraction and reverse transcription

Total RNA was isolated by use of TRIzol reagent (Invitrogen, Carlsbad, CA, USA) according to the manufacturers' recommendations. For paraffin-embedded tissues, sufficient sections were prepared, deparaffinised and conventionally re-hydrated in a decreasing alcohol-series prior to RNA extraction. Of the obtained RNA, one μg was reverse transcribed using the Reverse Transcription System (Promega, Madison, WI, USA). In order to improve transcription rate we mixed oligo-dT and pdN_6 _primers 1:2.

### Semiquantitative realtime PCR

Semiquantitative PCR was performed using the LightCycler system together with the LightCycler DNA Master SYBR Green I Kit (Roche Diagnostics, Mannheim, Germany) as described elsewhere [22]. To ensure experiment accuracy, all reactions were performed in triplicate. Primer sequences *were: FOXM1 *sense 5'-GAG CAC TTG GAA TCA CAG CA-3' and antisense 5'-CAC CGG GAA CTG GAT AGG TA-3'; *GAPDH sense *5'-GAA GGT GAA GGT CGG AGT CA-3' and antisense 5'-AAT GAA GGG CTC ATT GAT GG-3'. Annealing temperatures for both genes were set to 60°C. Reaction specificity was controlled by post-amplificational melting curve analyses as well as by gel electrophoresis of the obtained products.

### Immunohistochemical characterisation of the tissue microarray

Immunohistochemical (IHC) studies for the expression of HER2 utilised an avidin-biotin peroxidase method with a 3,3'-diaminobenzidine (DAB) chromatogen. After antigen retrieval (microwave oven for 30 min) IHC was carried out in a NEXES immunostainer (Ventana, Tucson, AZ, USA) following the manufacturer's instructions. The following primary antibodies were used: anti-HER2 (DAKO, Hamburg, Germany; 1:400), anti-ER and anti-PR (Novocastra, Newcastle Upon Tyne, UK; 1:20). For target proteins the ChemMate detection kit (DAKO) was used. A surgical pathologist (A.H.) performed a blinded evaluation of the TMA slides without knowledge of clinical data. Causes of non-interpretable results included lack of tumour tissue and presence of necrosis or crush artefacts. HER2 expression was scored according to the DAKO HercepTest. For the evaluation of ER and PR presence, a semiquantitative immunoreactivity score (IRS), as described by Remmele and Stegner [23], was used.

### FOXM1 Immunohistochemistry

The tissue microarray was subjected to immunostaining using the Advance Kit (DAKO K4068) following the manufacturer's instructions. Breast carcinomas were used as positive controls. After deparaffinisation and re-hydration the tissue samples were boiled in a microwave oven for 30 min in 10 mM sodium citrate buffer (pH 7.2). Endogenous peroxidase was blocked by Peroxidase blocking solution (DAKO S 2023). The polyclonal primary antibody FOXM1 (Santa Cruz sc502) was applied (1:30) for 1 h at room temperature. In negative controls the primary antibody was omitted. The Advance Kit HRP against rabbit/mouse (DakoCytomation K 4065, Carpintera, CA, USA) was applied to signal amplification for 30 min. For signal detection 3,3'-diaminobenzidine (DAB) was used. Slides were counterstained with hematoxylin and after dehydration mounted in Vitro-Clud (Langenbrinck, Emmendingen, Germany). An experienced pathologist (N.B.) scored the immunohistochemical staining intensity according to the scoring system suggested by Remmele and Stegner [23].

### Statistical analysis

For statistical evaluation the SPSS software version 14.0 (SPSS GmbH Software, Munich, Germany) was used. Differences were considered statistically significant when p-values were < 0.05. A non-parametrically two-tailed Mann-Whitney *U*-test was employed to analyse differences in expression levels. A statistical association between clinicopathological and molecular parameters was tested, using two-sided Fisher's exact test. Recurrence-free (RFS) and overall survival (OS) were calculated according to the Kaplan-Meier equation.

## Results

### Upregulation of FOXM1 in cancerous human epithelial breast cell lines

**Figure 1:**
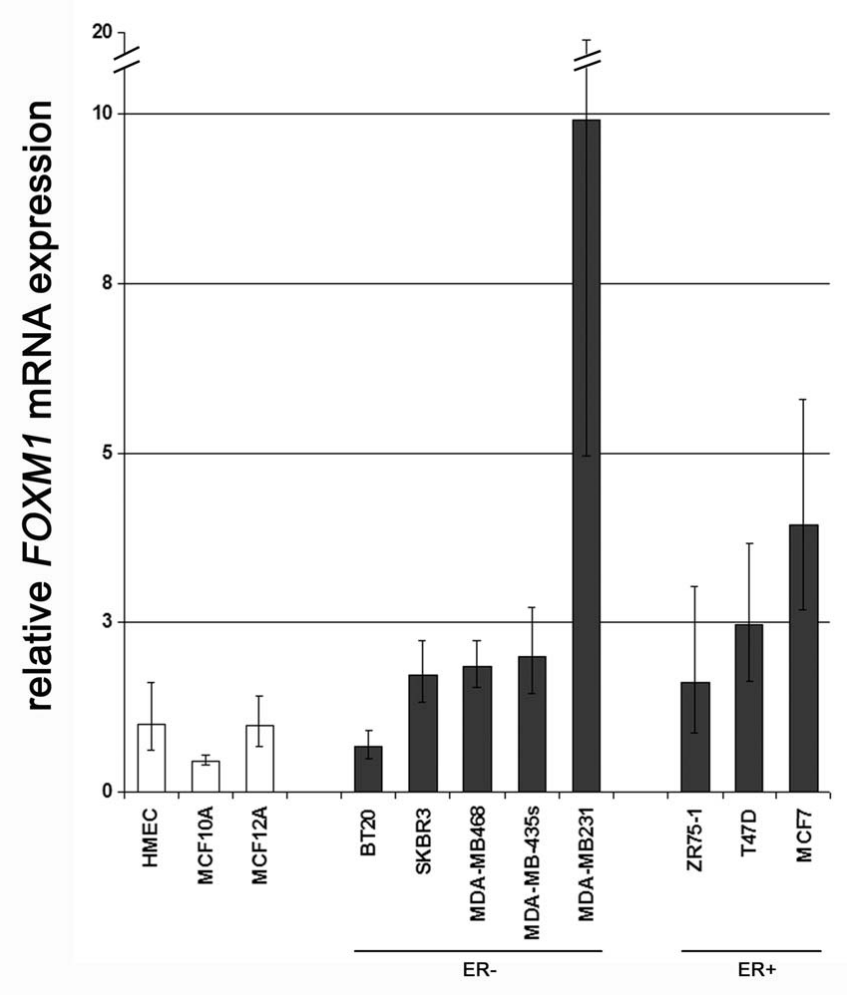
Elevated expression of *FOXM1 *mRNA in breast cancer cell lines. Semiquantitative realtime PCR (LightCycler) of *FOXM1 *expression was performed on reverse transcribed RNA from non-malignant (white bars) and malignant cell lines (black bars). A significant difference in expression between both groups was detected (*P *= 0.048), whereas classification of malignant breast cell lines into oestrogen receptor (ER) positive and negative revealed no significant coherence with *FOXM1 *expression (*P *= 0.655; two-tailed Mann-Whitney *U*-test).

We initiated our study by investigating the level of *FOXM1 *mRNA expression in non-malignant and malignant human mammary cell lines. Realtime PCR analysis revealed a homogenous *FOXM1 *mRNA expression in non-malignant mammary epithelial cell lines such as HMEC, MCF10A and MCF12A, whereas *FOXM1 *mRNA transcript levels were more heterogeneous and in total elevated in cancerous cell lines (Figure 1). The metastatic breast cancer cell line MDA-MB231 presented an exceptionally high level of *FOXM1 *expression (Fig. 1). The difference in *FOXM1 *expression between normal and cancerous breast cell lines was statistically significant (*P *= 0.048, Mann-Whitney *U*-test).

### Upregulation of FOXM1 in primary breast cancers

**Figure 2:**
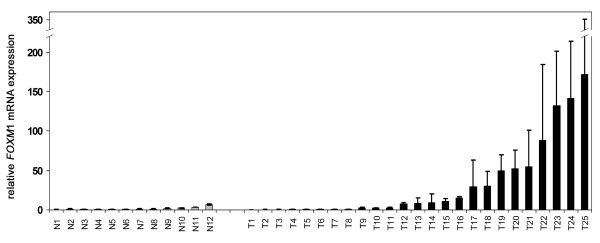
Upregulation of *FOXM1 *expression in primary breast cancer. A collection of paraffin-embedded breast carcinomas (T; n = 25) and normal breast tissues (N; n = 12) was analysed for *FOXM1 *expression by semiquantitative realtime PCR. In total, we detected a strong upregulation at transcript level in the tumourous tissues as compared with normal breast tissues.

Next we analysed *FOXM1 *mRNA expression by realtime PCR in primary human breast cancers (n = 25) and normal mammary samples (n = 12) derived from formalin-fixed, paraffin-embedded tissues. In line with the expression data in breast cell lines, *FOXM1 *mRNA was strongly upregulated in primary breast cancer specimens compared to normal mammary epithelial tissue (Fig. 2). The median fold change of *FOXM1 *upregulation in the cancerous tissue versus normal was 8.7 and difference in expression between the two groups was statistically significant (*P *= 0.029, Mann-Whitney *U*-test).

### FOXM1 protein expression in primary human breast cancer

**Figure 3:**
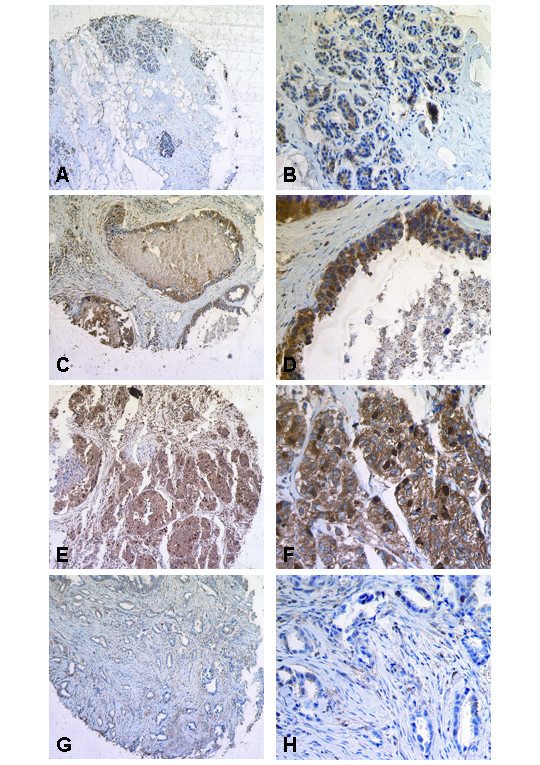
Immunohistochemical expression of FOXM1 in normal breast tissue as well as in non-invasive and invasive breast tumours using a tissue microarray. (A, B) Normal breast tissue is detected with very weak FOXM1 expression in the nucleus (IRS = 1) and weak FOXM1 expression in the cytoplasm (IRS = 4). (C, D) In ductal carcinoma *in situ *of high grade type nuclear FOXM1 expression is a little more intense (IRS = 2) than in normal breast tissue whereas the cytoplasmic FOXM1 expression is strong (IRS = 9). (E, F) In invasive breast carcinoma FOXM1 expression was often stronger in the nucleus (IRS = 3) as well as in the cytoplasm (IRS = 12) compared with normal breast tissue and ductal carcinoma *in situ*. (G, H) In tubular breast carcinoma, a rare variant of invasive breast carcinoma with a more favourable prognosis than invasive ductal breast carcinoma, nuclear and cytoplasmic FOXM1 expression (each IRS = 3) was weaker than in most invasive ductal breast carcinomas and stronger than in most normal breast tissues. Magnifications: A, C, E, G: 100×; B, D, F, H: 400×.

By use of a large tissue microarray (n = 250) we investigated the protein expression of FOXM1 in breast cancer specimens and normal breast tissues. FOXM1 is expressed in the nucleus as well as in the cytoplasm of benign and malignant mammary gland epithelial cells. Expression was absent in the fibrolipomatous stroma and in vessels. In normal breast tissue FOXM1 expression was often absent or weaker in the nucleus compared with breast carcinomas. In Figure 3(A, B) normal breast tissue is shown with very weak FOXM1 expression in the nucleus (IRS = 1) and weak FOXM1 expression in the cytoplasm (IRS = 4). In ductal carcinoma *in situ *of high grade type (Figure 3, C and 3D) nuclear FOXM1 expression is slightly more intense (IRS = 2) than in normal breast tissue whereas the cytoplasmic FOXM1 expression is strong (IRS = 9). In invasive breast carcinomas (Figure 3, E and 3F, ductal type) FOXM1 expression was often abundant in the nucleus (IRS = 3) as well as in the cytoplasm (IRS = 12) compared with normal breast tissue and ductal carcinoma *in situ*. In tubular breast carcinomas (Figure 3, G and 3H), a less frequent variant of invasive breast carcinomas with a more favourable prognosis than invasive ductal breast carcinomas, nuclear and cytoplasmic FOXM1 expression (each IRS = 3) was weaker than in most invasive ductal breast carcinomas and stronger than in most normal breast tissues. Considering the whole tissue microarray 87% (187/204) of the breast carcinomas expressed nuclear FOXM1 versus 42% (19/46) of normal breast tissue specimens. In accordance with this number the median nuclear immunoreactive score in breast carcinomas (median IRS = 3) was higher than in normal breast tissue (median IRS = 0).

### Correlation between nuclear FOXM1 and clinicopathological patient parameters

For statistical analysis only nuclear FOXM1 expression was considered because FOXM1 is known to be a transcriptional factor exercising its biological effect especially in the nucleus. Nuclear FOXM1 protein with an IRS ≥ 2 was considered as positive FOXM1 expression while IRS = 0 and IRS = 1 were considered as negative FOXM1 expression. Nuclear FOXM1 expression was not associated with tumour stage, lymph node status, histological grading, focality or histological type of tumour (Table 1). Nuclear FOXM1 was associated with the presence of oestrogen receptor with marginal significance (*P *= 0.085) but not with presence of progesterone receptor (*P *= 0.702). Importantly, a significant association was detected between nuclear FOXM1 and high expression of the HER2 receptor as determined by immunohistochemistry (DAKO score 3+; *P *= 0.045) (Table 1).

**Table 1:** Clinicopathological and immunohistochemical parameters in relation to nuclear FOXM1 immunoreactivity

**Variable**	**Categorisation**	**FOXM1 immunoreactivity**			
		**n analysable**	**negative**^**b**^	**positive**^**b**^	**p**^**c**^
***Clinicopathological data:***					
Tumour stage^a^					
	pT1	61	18	43	0.339
	pT2	96	27	69	
	pT3	14	1	13	
	pT4	28	9	19	
Lymph node status^a^					
	pN0	55	28	61	0.425
	pN1-3	139	27	78	
Histological grade					
	G1	22	10	12	0.146
	G2	90	25	65	
	G3	87	21	66	
Multifocality					
	unifocal tumour	174	48	126	0.820
	multifocal tumour	27	8	19	
Histological type					
	ductal	165	48	117	0.133
	lobular	15	1	14	
	other	18	6	12	
***Immunohistochemistry (IHC):***					
Oestrogen receptor status					
	negative	57	9	48	0.085
	positive	103	29	74	
Progesterone receptor status					
	negative	115	27	88	0.702
	positive	53	14	39	
HER2 status					
	weak (0–2+)	154	45	109	**0.045**
	strong (3+)	18	1	17	

^a^According to UICC: TNM Classification of Malignant Tumours. 6^th ^edn (2002) Sobin LH, Wittekind CH (eds) Wiley: New York

^b^FOXM1 nuclear immunoreactivity: negative = IRS 0–1, positive = IRS 2–12

^c^Fisher's exact test (two-sided), bold face representing significant data (*P *< 0.05)

**Figure 4:**
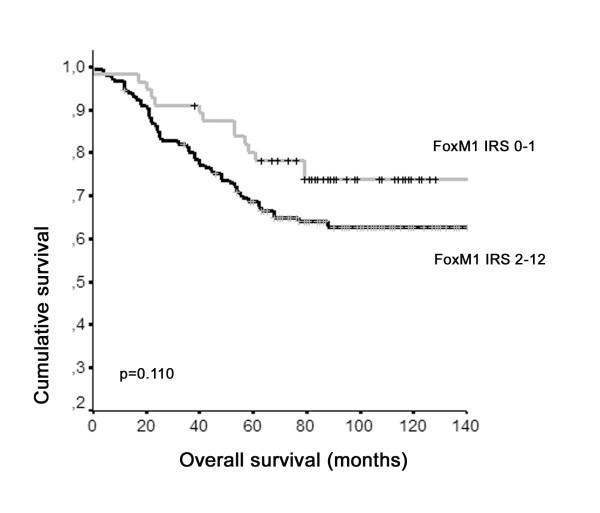
Breast cancer patients expressing nuclear FOXM1 show a tendency towards unfavourable prognosis. Univariate Kaplan-Meier analysis was performed on basis of expression results from a tissue microarray. Patients with weak or absent nuclear FOXM1 expression (IRS = 0–1) displayed improved overall survival estimation (upper graph) as compared to patients with strong nuclear FOXM1 expression (lower graph). Level of significance was only marginal (P = 0.110; univariate log-rank analysis). IRS=Immunoreactive score according to Remmele et al. [23]. Crosses indicate censored patients.

To investigate an impact of FOXM1 overexpression on patients' clinical outcome we calculated univariate survival probability curves with respect to immunohistochemical results. We found that nuclear FOXM1 expression in breast cancer (IRS ≥ 2) shows a tendency towards unfavourable prognosis regarding overall survival as shown by Kaplan-Meier analysis (*P *= 0.110) (Table 2, Figure 4). However, recurrence-free survival was not significantly associated with FOXM1 overexpression (Table 2).

**Table 2:** Univariate analysis of factors regarding overall survival (OS) and recurrence-free survival (RFS)

**Variable**	**Categorisation**	**Tumour-related death (OS)**			**Tumour recurrence (RFS)**		
		**n**	**events**	**p**^**c**^	**n**	**events**	**p**^**c**^
***Clinicopathological data:***							
Tumour stage^a^							
	pT1	61	11	**0.001**	59	14	**<0.0001**
	pT2	96	34		93	43	
	pT3	14	5		13	7	
	pT4	28	17		26	18	
Lymph node status^a^							
	pN0	89	15	**<0.0001**	87	17	**<0.0001**
	pN1-3	105	46		102	60	
Histological grade							
	G1	22	5	**0.003**	21	6	**0.001**
	G2	90	23		86	29	
	G3	87	39		85	46	
Multifocality							
	unifocal tumour	174	55	0.165	168	70	0.348
	multifocal tumour	27	12		25	12	
Histological type							
	ductal	165	53	0.333	162	70	0.494
	lobular	15	8		13	6	
	other	18	6		16	5	
***Immunohistochemistry (IHC):***							
Oestrogen receptor status							
	negative	57	24	**0.027**	57	29	0.057
	positive	103	27		99	33	
Progesterone receptor status							
	negative	115	49	**0.001**	109	52	**0.015**
	positive	53	8		53	14	
HER2 IHC							
	weak (0–2+)	142	42	0.055	135	53	0.120
	strong (3+)	31	14		31	16	
FOXM1^b^							
	negative (IRS 0–1)	56	14	0.110	54	23	0.581
	positive (IRS 2–12)	146	53		140	59	

^a^According to UICC: TNM Classification of Malignant Tumours. 6^th ^edn (2002) Sobin LH, Wittekind CH (eds) Wiley: New York

^b^FOXM1 nuclear immunoreactivity: negative = IRS 0–1, positive = IRS 2–12

^c^Log-rank test (two-sided), bold face representing significant data (*P *< 0.05)

## Discussion

Fox (Forkhead box) proteins are chordate transcription factors with an important role for cellular events such as regulation of cell growth and cellular development [1-3]. In accordance with the observed role of FOXM1 in cell cycle progression and cell proliferation, elevated expression of *FOXM1 *has been reported in several human tumour entities [7,8]. Recently, FOXM1 expression has been analysed in human breast carcinomas, showing that mRNA transcript levels of the *FOXM1 *gene were upregulated in tumour tissue as compared to normal breast tissue [7]. However, this study neither analysed FOXM1 protein expression in breast tumour tissues nor did it evaluate the impact of FOXM1 deregulation on patient prognosis. On a large cohort of breast cancer specimens we performed a first systematic expression analysis of FOXM1 on both mRNA and protein level and subsequently studied FOXM1 expression with respect to clinicopathological parameters and patients' survival data.

In concordance with the data from Wonsey et al. [7] upregulation of *FOXM1 *mRNA was found in paraffin-embedded breast carcinomas compared to normal breast tissue. Consistent with FOXM1 overexpression in human breast tumours, malignant mammary cell lines such as MCF7, MDA-MB231, MDA-MB468, T47D and BT20 also exhibited increased *FOXM1 *levels when compared to benign mammary cell lines (HMEC, MCF10A and MCF12A). Interestingly upregulation of *FOXM1 *was highly abundant in the breast cancer cell line MDA-MB231, a cell line which was shown to be strongly metastatic in nude mouse models [24]. This implicates a possible association between FOXM1 upregulation and enhanced proliferation and invasiveness of cancer cells.

Upregulation of *FOXM1 *mRNA in breast tumors could be confirmed on protein level when analysing the immunohistochemistry results from a tissue microarray. 87% of breast carcinomas were shown to exhibit nuclear FOXM1 staining, compared to a percentage of 41% of normal breast tissue specimens showing nuclear FOXM1 expression. Thus FOXM1 was shown to be upregulated on both protein and mRNA level. The median expression level in breast tumour tissue showed an IRS of 3 while the median IRS of normal tissue was 0. We next correlated nuclear FOXM1 expression with patients' survival data. Patients with nuclear FOXM1 expression showed a tendency towards unfavourable prognosis in overall survival analysis (*P *= 0.110). These data have to be confirmed in a prospective multi-centre trial including treatment information on FOXM1 positive and negative patients before FOXM1 overexpression in breast cancer can be considered as an established prognostic marker potentially useful for stratification of patient treatment.

This is the first study showing a significant association between nuclear FOXM1 expression and abundant expression of HER2 in breast cancer (*P *= 0.045). In a previous work of Yang et al. [25] FOXO4, another member of the Fox family, was found to inhibit HER2-activated cell growth by mediating p27 transcription. Therefore FOXO4 was discussed as a novel anticancer agent in HER2-overexpressing cancers. It may be concluded that FOXM1 and FOXO4 have opposite effects on *HER2 *gene expression. The human epidermal growth factor receptor 2 (*HER2*) gene is localised on chromosome 17q. It encodes a transmembrane tyrosine kinase receptor protein that is a member of the epidermal growth factor receptor (EGFR)/HER family [26]. *HER2 *gene amplification is found in 10–34% of invasive breast tumours and is regarded as an important prognostic marker indicating poor patient survival [27]. Thus determination of the HER2 status is a very important step in clinical routine to analyse the right therapeutic strategy. Intriguingly, HER2-positive breast carcinomas are often associated with negative oestrogen and progesterone receptor status [28]. So these breast carcinomas will not respond to treatment regimens solely based on anti-hormones. For this reason in a fraction of HER2-positive breast tumours a therapy with HER2 antibodies (Trastuzumab, Herceptin^®^) is applied. Nevertheless, most of the patients develop resistance to Herceptin^® ^during therapy [28]. Analyses of other potential target molecules in signalling pathways of malignant mammary cells are of great interest and may offer alternative treatment options for patients with Herceptin^® ^resistance. FOXM1 may be such a candidate molecule. Recently, FOXM1 inhibitors were discussed as new anticancer therapeutics [18,19]. Gusarova et al. [18] showed that inhibition of FOXM1 function by a cell-penetrating ARF (26–44) peptide lead to reduced tumour cell proliferation and angiogenesis in hepatocellular carcinoma. These studies were based on a mouse model. The apoptotic effect was also shown in human hepatoma cell lines. This inhibitory effect on FOXM1 has not been analysed for human breast cancer so far. Further functional studies including DNA binding studies are underway to analyse the interaction between FOXM1 and HER2, especially whether FOXM1 directly activates the *HER2 *(*c-erbB-2*) promoter. Recent studies show that FOXM1 protein might affect the transcription of oestrogen receptor alpha by binding to its promoter *in vitro *[10]. However, in our study we could not detect a significant correlation between FOXM1 expression and oestrogen or progesterone receptor status in human breast cancer. This might be related to the fact that previous studies were based on *in vitro *experiments using only breast carcinoma cell lines while our study analysed primary human cancer tissue. Considering the oestrogen receptor status we could in fact see a tendency towards negative correlation with nuclear FOXM1 expression (*P*= 0.085). As HER2-positive breast carcinomas are often associated with negative oestrogen and progesterone receptor status [28] these findings are in concordance with the results from our immunohistochemistry data.

Taken together, our analyses showed FOXM1 overexpression in human breast carcinoma relative to normal breast tissue on RNA and protein level indicating that FOXM1 may represent a novel oncogene in human breast cancer development. Interestingly, we could find a positive correlation between FOXM1 expression and HER2 status pointing to the potential role of FOXM1 as a new drug target in HER2 resistant breast tumour. Further studies are underway to analyse the interaction between FOXM1 and HER2, especially whether FOXM1 directly activates the *HER2 *promoter and thus inevitably leads to HER2 overexpression.

## Conclusion

FOXM1 may represent a novel breast tumour marker with prognostic significance that could be included into multi-marker panels for breast cancer detection and treatment. Interestingly, we found a positive correlation between FOXM1 expression and HER2 status, additionally pointing to a potential role of FOXM1 as a new drug target in HER2 resistant breast tumour, as FOXM1 inhibitors for cancer treatment were recently described. Further studies are underway to analyse the potential interaction between FOXM1 and HER2, especially whether FOXM1 directly activates the *HER2 *promoter.

## Abbreviations

FOXM1: Forkhead transcription factor; ER: Oestrogen receptor; *ERS1*: Oestrogen receptor gene 1; PR: Progesterone receptor; *GAPDH*: Glyeraldehyde-3-phosphate dehydrogenase; H&E: hematoxylin and eosin; FISH: fluorescent *in situ *hybridisation; ATCC: American Type Culture Collection.

## Competing interests

The author(s) declare that they have no competing interests.

## Authors' contributions

NB: participated in design of the study, data analysis, data interpretation, establishment and evaluation of the immunohistochemistry and drafted the manuscript; AtH: carried out the immunohistochemical studies, and critically revised the manuscript; JV: supported with expertise in molecular biology techniques and in data interpretation and critically revised the manuscript; PJW: supported in data interpretation and critically revised the manuscript; JLF: supported in establishment of the immunohistochemistry, data interpretation and critically revised the manuscript; RK: participated in design and coordination of the study, and critically revised the manuscript; ED conceived the study, participated in study design and coordination, molecular and data analysis, data interpretation and drafting of the manuscript.

All authors read and approved the final manuscript.

## Pre-publication history

The pre-publication history for this paper can be accessed here:


